# Air Pollution Control and Public Health Risk Perception: Evidence from the Perspectives of Signal and Implementation Effects

**DOI:** 10.3390/ijerph19053040

**Published:** 2022-03-04

**Authors:** Yangyang Fan, Liangdong Lu, Jia Xu, Fenge Wang, Fei Wang

**Affiliations:** 1 School of Management, University of Science and Technology of China, Hefei 230026, China; fanyy@mail.ustc.edu.cn; 2 Business School, Hohai University, Nanjing 210098, China; luliangd@ustc.edu.cn; 3 Water Resources Management Office, Zhangjiagang Water Conservancy Bureau, Suzhou 215638, China; mico131220@sina.com; 4 International School of Business & Finance, Sun Yat-sen University, Guangzhou 510275, China; wangf289@mail.sysu.edu.cn

**Keywords:** air pollution control, public health risk perception, signal effect, implementation effect

## Abstract

The main purposes of government environmental policy include improving the objective natural environment as well as reducing the health risk of the public. A majority of studies have tested the means of achieving the first goal. In this paper, we aimed to gather empirical evidence pertaining to the realization of the second goal by drawing on a quasi-natural experiment that was conducted based on the “Action Plan on Air Pollution Prevention and Control” issued in 2013 (AP2013). The research data came from the tracking data of 17,766 individuals from 112 prefecture-level cities of China in 2012 and 2014. Through ordinal logistic regression and DID analysis, a causal relationship between the AP2013 policy and public health risk perceptions was verified, indicating that this policy can significantly decrease public health risk perceptions. By constructing different subsamples, an inverted U-shaped relationship between the causal effect and the length of the policy implementation window was established, which demonstrated the short-term signal effect and long-term implementation effect of this policy. The conclusions can help with the communication and implementation of a government’s policy.

## 1. Introduction

One of the main objectives of government policymaking is regulating public expectations and behaviors [1]. Government policy can be regarded as a gradual process from formulation to promulgation, integrating experience and knowledge, current problems, and social and public expectations [2], aiming to solve the problems facing or to be faced by the public. China’s rapid development of the economy has resulted in a large range of factors contributing to environmental pollution in many cities, which has seriously affected the health and happiness of the public.

To solve this problem, a number of environmental policies have been issued by China’s State Council and local governments. On September 10, 2013, to address the regional atmospheric environmental problems characterized by inhalable particulate matter (PM10) and fine particulate matter (PM2.5), China’s State Council promulgated the “Action Plan on Air Pollution Prevention and Control” (AP2013) and issued ten measures for the prevention and control of air pollution. Since then, local governments have successively issued local air pollution prevention and control policies. Thus, AP2013, the landmark policy of environmental governance in China, has been implemented in many regions, providing us with a good opportunity and an excellent research window to study the impact of environmental policies on public health risk perceptions.

The main actions of AP2013 are to strengthen the comprehensive treatment of pollution and reduce the emissions of multiple specified pollutants; optimize the industrial structure, while promoting industrial transformation and upgrading industry; accelerate the adjustment of China’s energy structure and increase the supply of clean energy; clarify the responsibilities of the government, enterprises, and society to mobilize the whole population to participate in environmental protection. The specific target was to reduce the concentration of inhalable particulate matter in cities at and above the prefecture level by more than 10 percent by 2017, with the number of fine days increasing year by year. In particular, the concentration of fine particulate matter in the Beijing–Tianjin–Hebei region, Yangtze River Delta, and Pearl River Delta were to decrease by about 25%, 20%, and 15%, respectively.

Several studies have attempted to demonstrate the effects of AP2013 on China’s society and economy, as it is known as the most stringent action plan for air pollution control ever in China. M. Li et al. (2020) found that this policy had a significant negative impact on the total factor productivity of regulated enterprises in A-share listed companies, and the stock performance of polluting industries was significantly lower than that of other industries [3]. The implementation of the policy greatly improved air quality and public health, a fact which has been empirically verified by many researchers. From 2013 to 2017, the implementation of AP2013 significantly reduced the air pollution in cities across the country and eliminated more than 50% of the winter pollution problems caused by heating in northern China [4]. By 2017, the PM10 concentration in 338 cities had decreased by 22.7% on average compared with 2013, and the PM2.5 concentration in 74 key cities decreased by 34.3% overall [5]. The monthly average emissions of PM2.5, PM10, sulfur dioxide, nitrogen dioxide, carbon monoxide, and other pollutants in the Beijing–Tianjin–Hebei region, Yangtze River Delta, Pearl River Delta, and other regions were significantly reduced [6,7,8,9]. According to research on Beijing, from 2013 to 2017, the emissions of the pollutants PM2.5, PM10, nitrogen dioxide, and sulfur dioxide decreased by 34%, 24%, 17%, and 68%, respectively [10,11]. The benefits coming from reducing losses to human health, agriculture, building materials, and cleaning in Beijing increased from CNY −1.928 billion in 2013 to CNY 22.33 billion in 2017 [12]. According to an estimate of Peking University, the improvement of air quality brought about by the implementation of AP2013 reduced the death toll in 74 Chinese cities by 47,240 in 2017 compared with 2013 [13].

However, as a policy that aims to improve the living environment of the whole society, few studies have paid attention to its impact on public health awareness. This paper seeks to remedy this issue. Previous research has found that there are many factors affecting public health risk perceptions. Epidemiological data analysis shows that education level, income, age [14,15], working environment [16], recreational and sports activities [17], gender, foreign language proficiency [18,19], nutrient level [20], smoking habits, and objective physical health conditions, such as cardiovascular diseases [21], are all related to health awareness. The pollution of the surrounding environment has also been proven to be an important factor that has a serious impact on the public’s perception of their health. The public’s perception of their health is deteriorated by exposure to organochlorine pollutants and dioxins in the working environment [22]; pests, molds, and poor ventilation found in the family living environment [23]; the use of fire in a home stove [24]; and particulate pollutants in the air [25]. Air pollution with an excessive PM2.5 concentration harms public mental health [26].

The promulgation of the government’s environmental policy may affect public health risk perceptions through two channels: the signal released by the policy and the actual effect produced by the policy. These, respectively, are called the policy signal effect and policy implementation effect in this paper. The signal effect means that the government sends signals to the market and the public through intervention to achieve a certain goal. The fundamental concern of the signal effect is to reduce information asymmetry between the government and the public. The policy signal effect is reflected in the environmental governance policy issued by the government, which transmits to the public the signal that the government is about to strictly control air pollution, making the public increase their expectations of environmental improvement and health improvement, which is expected to reduce the public’s perception of health risks. The policy implementation effect is reflected when the environmental policy issued by the government effectively changes the environmental situation, and the public feels the improvement of the environment, thus lessening the public’s perception of health risks. Since Spence (2002) put forward the signal effect theory [27], the signal effect has been studied in many fields, such as corporate governance [28], innovation and entrepreneurship [29], venture capital [30], and human resource management [31]. However, there is no relevant research in the field of environmental policy. In this paper, we study the policy signal effect of AP2013 on public health risk perceptions and its differences from the implementation effect of that policy.

The main innovations of our study are as follows. A quasi-natural experiment is constructed based on AP2013 to study the causal relationship between government environmental policies and public health risk perceptions. From the perspectives of the policy signal effect and policy implementation effect, this paper analyzes the inverted U-shaped relationship between the length of the policy implementation time window and public health risk perception. Based on two-period tracking data and combined with the Difference-in-Differences (DID) method, the research conclusion is accurate and robust.

The paper is organized as follows. Section 2 describes the methodological framework. Section 3 presents the empirical results of several versions of the function. Section 4 concludes the paper.

## 2. Methodological Framework

### 2.1. Data and Sample

In this research, 112 prefecture-level cities in 29 provinces and autonomous regions in China in 2012 and 2014 are selected as research samples. We use the China Labor-force Dynamics Survey (CLDS), which is a large-scale continuous nationwide research project planned by Sun Yat-sen University and implemented by the Social Science Survey Center of Sun Yat-sen University. The purpose of the research project is to systematically collect information about China’s labor force through regular tracking, summarize the long-term trends of social changes, discuss issues of major theoretical and practical significance, and provide information for government decision making and international comparative research. CLDS provides three levels of sample surveys about the labor force: the individual level, family level, and village level. Detailed questions are set in the questionnaire for each level. This paper mainly uses the survey results from the individual level and family level. At the level of the individual laborer, questionnaires are set to glean the aspects of basic information, educational experience, occupational status, nonagricultural work history, job status and participation process, social participation and support, labor status, reproductive history, and health status. At the family level, questionnaires are used to gather aspects of basic information, daily life, housing conditions, family economy, floating population family, agricultural production, family history, and the occupations of family members. When using CLDS data, this paper mainly uses the panel data for 2012 and 2014 and obtains individual and family tracking data of the same labor force in 2012 and 2014 by tracking individual identification numbers.

The urban control variables used in this paper are from the China City Statistical Yearbook. China’s National Bureau of Statistics conducts a survey every year to assess urban development, and the China City Statistical Yearbook contains detailed statistical data on the social and economic development of 658 Chinese cities, including administrative divisions, population, labor force, comprehensive economy, industry, transportation, post and telecommunications, education, etc., comprehensively reflecting the social and economic development of Chinese cities.

In response to air pollution, China’s State Council and local governments at all levels have promulgated various policies to control air pollution. On 27 September 2012, China’s State Council approved the “12th Five-year Plan for Air Pollution Prevention and Control in Key Areas” (referred to herein as the Plan). The Plan delimited 13 key areas for air pollution prevention and control, including the Beijing–Tianjin–Hebei, Yangtze River Delta, Pearl River Delta, Jiangsu urban agglomeration, and Shandong urban agglomeration areas, setting governance and improvement objectives for problems of high concern, such as PM2.5 and haze weather. Many aspects of the Plan are the same as AP2013, such as optimizing the energy structure and the control of coal use, improving the way coal is used and promoting the clean use of coal, strengthening the control of industrial smoke and dust, and vigorously reducing the emissions of particulate matter. The key measures to control air pollution in the Plan are also the key policies of AP2013. Therefore, referring to the research of Li Haoran (2018), this paper regards the release time of the Plan as the release time of AP2013 [4].

We determined the specific implementation time of AP2013 (the Plan) in 112 cities; the implementation times in some cities are shown in Table 1 as examples. The implementation time of AP2013 varies from October 2012 to October 2014 in different cities. In this paper, the dummy variables for AP2013 are generated according to the time interval of the implementation time of AP2013 in each city and questionnaire survey.

In the final dataset, 17,766 individual tracking data are obtained from 29 provinces (including autonomous regions), 112 prefecture-level cities, and 11,090 families in mainland China. The dataset omits information for Hong Kong, Macao, Taiwan, Tibet, and Hainan due to a lack of CLDS data.

### 2.2. Methodology

From 2012 to 2014, public health risk perceptions changed because of many factors. In this paper, we study the effect of policy treatment, that is, the change in public health risk perceptions caused by the implementation of AP2013. This study uses the phased implementation characteristics of AP2013 in various cities, takes this exogenous event as a quasi-natural experiment, and clarifies the causal relationship between the AP2013 environmental policy and public health risk perceptions. The basic idea is this: on the one hand, the health risk perception of the same individual changed from 2012 to 2014; on the other hand, at the same time, differences appear in health risk perceptions between individuals in cities with and without the implementation of AP2013, and the Difference-in-Differences method (DID) can effectively identify the effect of policy treatment. By constructing the experimental group and the control group, the DID model compares the difference between the average change of the experimental group from 2012 to 2014 and the average change of the control group to test the treatment effect of the policy. As an exogenous event, AP2013 provides a quasi-experimental opportunity to use DID to solve the endogeneity problem. The experimental group of this study is the public of cities that implemented the AP2013 policy one month before the 2014 questionnaire survey, and the control group is the public of cities that did not implement the AP2013 policy before the 2014 questionnaire survey.

This study uses various control variables and individual fixed effects to control the differences among different individuals. Since the dependent variable to be explained is a categorical variable taking a value from 1 to 5 (see Section 2.3.1), we use ordinal logistic DID regression to test the impact of the implementation of AP2013 on public health risk perceptions. The baseline DID model is as follows:

(1) $$PHRP_{ict}=\alpha +\theta Time_{t}*Airten_{ict}+\gamma Time_{t}+\beta Airten_{ict}+\delta X_{ict}+\mu_{i}+\varepsilon_{it}$$

where the dependent variable, $PHRP_{it}$, is the public health risk perception of individual i in city c at year t.$\;Time_{t}$ is the time dummy variable. $Airten_{ict}$ is the dummy variable of AP2013, which refers to whether the city *c* that individual *i* is in has implemented the AP2013. $Time_{t}*Airten_{ict}$ is the cross term of the time dummy variable and AP2013 dummy variable, which is the core explanatory variable of concern in this study. Finally, $X_{ict}$ represents the control variables, and $\mu_{i}$ is the individual fixed effect.

### 2.3. Variables

#### 2.3.1. Dependent Variable: Public Health Risk Perceptions

This study measures Public Health Risk Perceptions (PHRP) at the individual level of the labor force, with data from the China Labor-Force Dynamics Survey (CLDS). The health risk perception data for this study comes from the following item in the questionnaire: “What do you think of your current health?” The options are: 1. Very healthy, 2. Healthy, 3. Average, 4. Unhealthy, 5. Very unhealthy.

According to the setting of the questionnaire, the higher the score, the stronger the health risk perception of the individual laborer.

#### 2.3.2. Independent Variables

This study uses dummy variables for AP2013. In this study, the time difference between AP2013 and CLDS is used as the basis for the division of the experimental group and control group. The implementation time of CLDS is from June to August 2012 and from June to August 2014. The survey time of each labor force can be specific to a certain month. If the implementation time of AP2013 is one month before the CLDS in the city where a labor force is located, that is, if the time window is more than one month, then the city is considered to have implemented AP2013 and is classified as the experimental group and the Airten variable set as 1; otherwise, it is classified as the control group, with the Airten variable as 0.

Dummy variables are also used for time variable. CLDS was conducted in 2012 and 2014. Because China’s society has changed rapidly, the public’s health risk perceptions also change significantly with time, so a time variable is used in this analysis. If the sample is from 2012, Time is taken as 0; otherwise, it is 1.

#### 2.3.3. Control Variables

The control variables are implemented at the microscopic level. Studies have shown that people of different ages have a large difference in their perception of health risk [15], and that economic conditions have a significant impact on public health perceptions [14], and better and more convenient medical conditions [32] have improved the level of health services and their own health. Therefore, this study used individual age, annual household income, and distance from the home to the nearest health point to control for age, economic, and health factors at the microscopic level.

Control variables are also present at the city level. Regional industrial development level and regional pollution [22,33] also have a certain correlation with public health risk perceptions. Therefore, this paper adds the proportion of the output value of the secondary industries in GDP (i.e., the proportion of industrial output value in GDP) and the comprehensive utilization rate of urban industrial solid waste as urban control variables.

#### 2.3.4. Statistical Analysis

According to the descriptive statistical analysis results of full-sample variables in Table 2, the mean value of PHRP is 2.455, which was intermediate between “Average” and “Healthy” in the corresponding questionnaire. This level indicates that public health problems were prevalent from 2012 to 2014, which was consistent with the overall development of China at the time.

## 3. Empirical Results

### 3.1. Results of Ordinal Logistic Regression Analysis

Before the regression analysis, a collinearity test and parallelism test were performed to ensure that the ordinal logistic DID equation (1) is suitable for this case. The results of the basic analysis are shown in Table 3. For the PSM matched sample data, when the control variables are not considered, the partial regression coefficient of Time*Airten is negative, indicating the trend that the AP2013 has significantly decreased public health risk perceptions. The results remain significant after controlling for the microscopic and city control variables, with a coefficient of −0.122, and the odds ratio (OR) is 0.885. At the same time, comparing the regression analysis results of the PSM matched and unmatched data in Table 3, we can find that the Time*Airten coefficients of all models in Table 3 are negative. The analysis results show that the effect of AP2013 on the overall health risk perceptions of the public is significant.

In addition, the regression results of the control variables are also consistent with our expectations. From the coefficient of the control variables, we know that with the growth of age, the health risk perception of an individual generally increases, and the increase in family income can significantly reduce the public’s health risk perceptions. The farther the nearest medical service from the family is, the higher the public’s perception of health risk is. The level of urban industrialization shows the development level of the city. From the results, the higher the level of urban industrialization and the higher the comprehensive utilization rate of urban industrial waste, the smaller the public’s health risk perceptions.

### 3.2. The Analysis of the Mechanism of the Policy

For the experimental group for the previous results, we selected the public in cities where AP2013 had been implemented for at least one month before the questionnaire survey in 2014. In this section, we select different time window periods, from 1 month to 6 months, to generate other experimental groups, respectively denoted as “window 1” to “window 6”, to analyze the effects of AP2013 at different times. Our base case of one month is already performed as window 1. The people in cities that did not implement the AP2013 before the survey continue to be the control group, which together with the experimental group constitute a subsample.

**Figure 1:**
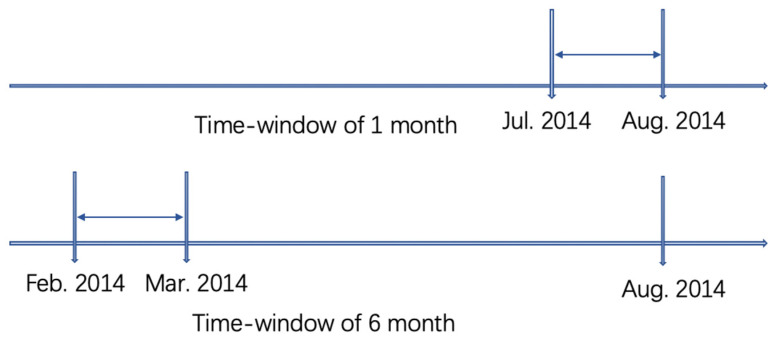
Schematic diagram of generation of 1-month time window and 6-month time window experimental group.

Taking the 1-month and 6-month time windows as examples, we will explain how to generate subsamples of the experimental group. Assume that the focal individual was surveyed in August 2014. If the implementation of AP2013 in the individual’s city is after August 2014, the individual will be classified as the control group, and the value of Airten is 0. As shown in Figure 1, if the individual was surveyed in August 2014 and the individual’s city implemented AP2013 in July 2014, then the individual will be classified into the experimental group in the 1-month time window subsample, and Airten is taken as 1. If the implementation time of AP2013 of the city is some other time, the data will be discarded.

As shown in the other part of Figure 1, if the individual’s city implemented AP2013 in February 2014, the individual will be classified into the experimental group of the 6-month time window subsample, and Airten is taken as 1; if the implementation time of AP2013 in that city was another time, these data are discarded.

**Figure 2:**
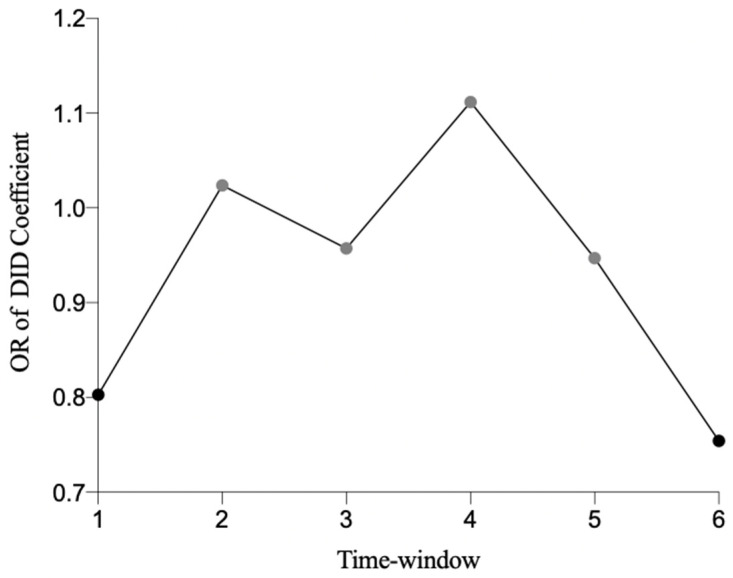
Ordinal logistic regression results of different time windows.

According to the results in Table 4, in the short term (one month) and long term (six months) after the promulgation of the environmental policy, the public’s perception of health risk significantly decreased. The OR of Time*Airten was calculated, and the results of each time window were plotted, as shown in Figure 2. In a period of time after the promulgation of AP2013, the public perception of health is an inverted “U” shaped curve. There is a transition period of two to four months after the promulgation of the environmental policy in which the public’s perception of health risk increases, although the coefficient is not significant.

The results for window 6 shows that the Time*Airten coefficient is significantly negative, with the OR equal to 0.754. This indicates that there is a long-run effect of the policy (i.e., six months after the promulgation of the AP2013 policy). That is, the significantly improved air pollution at that time, which has been found in the research of Zhang et al. (2016) [34], can reduce the public’s health risk perceptions. Compared with the signal effect conveyed by the policy, the actual improvement of the environment brought by the policy has a stronger encouraging effect on the public, which we know from the analysis result that the policy result effect (0.754) is stronger than the policy signal effect (0.803).

Two to four months after the promulgation of the AP2013 policy, the public’s perception of health risks ascended. Wei, Zhao, and Liang (2009) put forward three information diffusion models [35]. We believe that the information diffusion of the policy follows the decreasing information diffusion model, so it will take some time for AP2013 to come into effect from promulgation to implementation, and more time still for the impact to be felt. In this process, the effect of information diffusion gradually diminishes, and the policy signal effect weakens gradually. At that point, the policy has not brought significant improvement to the environment yet, causing the public’s health risk perception to improve, reaching a peak of 1.111 four months later. Then, the actual effect of the policy gradually emerged, and the public’s health risk perception gradually decreased.

### 3.3. Heterogeneity Analysis

Due to the externalities of air pollution, it has different effects on different regions and different groups of society. We explore the heterogeneous impact of the AP2013 policy on public health risk perceptions from three aspects: region, gender, and age.

Here, we analyze regional differences. The State Council of China divides the central, western, eastern, and northeastern economic regions according to the level of economic development, our analysis is based on this division, of which the northeast region is discarded due to a small amount of data. It can be seen from the results in Table 5 that in the western, central, and eastern regions of China, the AP2013 has played a role in determining public health risk perceptions. The policy effect in the eastern region is greater than that in the central and western regions, and the effect is not significant in the western region, showing that the public in the western region is relatively less affected by this policy. From the regression results of the subsample, the short-term policy effect in the central region was significant (column 5), while the long-term policy effect in the eastern region was better (column 9). The main reason may be that the central region is more sensitive to policy signals, and the long-term policy implementation effect in the eastern region is better.

Here, we analyze the gender differences. From the results in Table 6, it can be seen that the Time*Airten regression coefficients of both men and women in the whole sample are negative, indicating that AP2013 has lowered the perception of health risks in men and women. The significance of the female sample is better than that of men, and the policy effect coefficient is larger, indicating that the effect of the policy on women is more obvious. At the same time, from the subsample analysis, the short-term and long-term influence on women of the policies is significant, while the short-term influence on men of the policies is more significant and the coefficient is greater than that of women, but the long-term impact is not significant. From a physiological point of view, women’s lung tissue and trachea are smaller and more sensitive to the effect of improving air quality, while women’s mood fluctuates greatly due to changes in hormone levels [36], so it is more significantly affected by the long-term implementation of policies. From a psychological point of view, men are more sensitive to political and national policies [37] and are, therefore, more significantly influenced by the short-term signaling effects of policies.

Here, we analyze age differences. Considering the maturity of individual perceptual ability, this part of the test selected a sample of adults aged over 18 years old, and divided the samples into middle and young age segments (18–55 years old) and senior age groups (over 55 years old). Judging from the regression results in Table 7, the AP2013 had a more significant effect on the health risk perceptions of people over 55 years old, especially in the subsample of the 6-month time window, where the policy effect coefficient of people over 55 years old is −0.821, with the OR equal to 0.440. In the long run, the implementation of AP2013 has significantly and vigorously decreased the health risk perceptions of the elderly population.

### 3.4. Robustness Check

To test the reliability of the conclusions, robustness tests, such as propensity score matching, removing certain values of control variables, and replacing control variables, were carried out.

The DID method can partially solve the problem of sample endogeneity, but it cannot solve the problem caused by sample selection deviation. We use propensity score matching (PSM) to solve this “selection” problem. Before using the DID model shown above, PSM is used to match the treatment group with the corresponding control group. The specific process is as follows. First, we select the relevant urban variables and the control variables in the model as the covariates. These include the north and south of the city, the total population at the end of the year, land area, per capita GDP, hazard-free treatment rate of urban waste, annual family income, and other variables. Next, the propensity score is calculated using the logit model. The nearest neighbor matching, kernel matching, and radius matching methods are used to select one-to-one matching samples from the control group for each person in the treatment group. We then test the balance of the matched samples and delete inconsistent data. After completing the above matching process, we used the model equation for the regression analysis again. The results are shown in Table 8, showing that the results have not changed substantially, showing the robustness of our conclusions.

In the meantime, a few extreme values in the control variables may greatly impact the regression results. For example, the median annual household income is CNY 30,000, and the average is CNY 48,474.29, while some annual household incomes are more than CNY 1,000,000, and the maximum value is CNY 6,000,000. After Winsorizing the variables of the data, we performed the regression again, finding that the coefficient of the core variable shown in Table 9, column 1 is still significantly negative, indicating that the research conclusion of this paper is robust.

Next, we replaced the control variables in the model equation (in Table 9, column 2), substituting variables with similar meanings, such as replacing the annual household income with the urban per capita GDP, the number of doctors per 10,000 people in cities replacing the distance to the family’s nearest medical point, and the proportion of tertiary industry output value in GDP replacing the proportion of industrial output value in GDP. Then, we conducted the regression again, and found that the core variable coefficient remains significantly negative, showing the reliability of the conclusion. Considering that the public’s risk perception may also be influenced by factors such as educational experience, living environment, work status, and unexpected events (such as being assaulted, cheated, stolen), we conducted a further robustness test after controlling for these factors. As can be seen from the regression results in Table 9, column 3, the conclusions are robust and unchanged.

## 4. Discussion

### 4.1. Research Contribution

As mentioned above, reducing residents’ health risk perceptions is an effective way to improve people’s happiness and security. Since 2017, the Chinese government has proposed to strive to make the fruits of reform and development benefit all the people, and continuously enhance the people’s sense of gain, happiness, and security. It shows that the government’s governance incorporates the subjective perception of the public as an important goal of governance, but there is a lack of policy evaluation research aimed at the subjective perception of the public in the current research. We study the effect of China’s environmental policy from the perspective of public health risk perceptions, which is a good supplement to the research on the objective improvement due to environmental governance. Secondly, based on the quasi-natural experiment provided by the implementation of AP2013, combined with the data of China’s labor force survey database for 2012 and 2014, the research conclusion has a strong causal inference and empirically tests the causal relationship between environmental policy and health risk perceptions. Finally, we infer the internal mechanism of how environmental policy affects public health risk perceptions by analyzing the inverted U-shaped relationship between the length of the environmental policy implementation window and public health risk perceptions, using the perspectives of policy signal effect and policy implementation effect. This study shows that the government’s air pollution control policy can improve the public’s subjective perception of sense of gain and happiness, and we also put forward policy suggestions that attach importance to short-term perceptions and long-term perceptions, which has certain practical significance for China’s governance. In addition, in view of the differences in the perception of different groups, targeted measures need to be taken in the process of policy implementation. For example, men are more sensitive to policy signals than women, and women are more responsive to substantial improvements in the atmosphere. This requires integrated advocacy and the implementation of environmental policies, with targeted measures for people of different ages, genders, and regions.

### 4.2. Deficiencies and Prospects

The survey data of China’s labor force used in this paper may have sample deviation. Although the survey team adjusted the sampling probability during data collection and ensured that the sample had the broadest practical coverage, the subjective evaluation of the labor force on their health status is easily affected by the time and events around the time of the questionnaire survey. For this reason, there may still be confounding deviations in the questionnaire results.

In our study, two periods of panel data are used to analyze the impact of government environmental policies on public health risk perception. The panel data of the two periods have certain defects in policy evaluation. For example, the two periods of panel data did not allow us to carry out a parallel trend analysis with the DID analysis. The data used in this paper cover two periods of tracking, and the PSM method is adopted to balance the samples of the processing group and the control group, which alleviated this defect to a certain extent. Although we were able to find the impact of two different effects on public health risk perception, more comprehensive data may bring further results.

When analyzing the subsamples generated in different time windows, we infer the role of the policy signal effect and policy implementation effect on health risk perceptions. From the perspective of signal theory, the signal effect of the policy shows a monotonous decreasing trend [35], so whether the impact of policy on the public follows the inverted U-shape law mainly depends on the time when the effect of the policy is generated. For public policies that require a certain period of time to produce policy effects, we believe that they all follow the U-shaped law; however, for policies that will have an immediate effect after implementation, they may not have a U-shaped effect, such as environmental taxation, carbon emission pricing policy. However, we do not provide strong empirical analysis support for this inference. The connection and connection mechanism between policy signal effect and policy implementation effect can be a subject of further research in the future.

## 5. Conclusions

As the most stringent environmental policy to date in China, AP2013 has had a profound and significant impact on the public’s perception of health risks since its promulgation and implementation in 2013. Based on dynamic survey data of China’s labor force in 2012 and 2014 from 29 provinces and autonomous regions, and considering the difference in AP2013 implementation times in different cities, we obtained two periods of individual tracking data. Considering AP2013 as an exogeneous policy, we used the ordinal logistic PSM-DID method to control for variables such as age, economic conditions, medical and health level, and urban pollution status. We find that the implementation of AP2013 significantly decreased the public’s perception of health risks. By constructing subsamples of different policy implementation time windows, the study further finds an inverted U-shaped relationship between the implementation time of the government’s environmental policy and public health risk perceptions.

Our study explains the conclusion from two aspects: the policy signal effect and policy implementation effect. At the beginning of the implementation of environmental policies, the public’s expectations rapidly rise due to the signal effect, significantly reducing the public’s perception of health risks. With diminishing information diffusion, the signal effect of the government environmental policy decreases gradually, and the public’s health risk perception begins to grow, reaching the peak in the fourth month after implementation. Then, the actual effect brought about by the policy implementation begins to appear, the public’s health risk perception begins to descend, and the descension brought about by the policy implementation effect is stronger than the change brought by the policy signal effect.

## Figures and Tables

**Table 1:** Implementation time of AP2013 in some cities.

City Name	Time	City Name	Time	City Name	Time
Beijing	Sep. 2013	Xuzhou	Jun. 2014	Luoyang	Jan. 2014
Tianjin	Sep. 2013	Yangzhou	May 2014	Pingdingshan	Sep. 2014
Baoding	Sep. 2013	Hangzhou	May 2014	Shangqiu	Jun. 2014
Xingtai	Nov. 2013	Jiaxing	Apr. 2014	Xinyang	May 2014
Langfang	Sep. 2013	Ningbo	Jun. 2014	Xuchang	Dec. 2014
Tangshan	Oct. 2013	Taizhou	May 2014	Zhengzhou	May 2014
Zhangjiakou	Oct. 2013	Wenzhou	Apr. 2014	Wuhan	Feb. 2014
Taiyuan	Oct. 2013	Hefei	May 2014	Jinmen	Oct. 2014
Linfen	Oct. 2013	Huainan	Mar. 2014	Jingzhou	Nov. 2014
Jincheng	Nov. 2013	Liuan	Mar. 2014	Xianning	Feb. 2014
Ulanqab	Nov. 2013	Xuancheng	Feb. 2014	Huanggang	Feb. 2014
Jinzhou	Jun. 2013	Bouzhou	Mar. 2014	Changsha	Mar. 2014

**Table 2:** Descriptive statistical analysis of variables.

Variables	Meaning	Number of Samples	Mean	Standard Deviation
PHRP	perception of health risk	17,766	2.455	0.99
Time	dummy variable for time	17,766	0.500	0.5
Airten	dummy variable for AP2013	17,766	0.610	0.48
Age	age	17,702	45.69	12.84
AHIncome	annual household income (CNY)	16,980	48,474.29	107,388.7
DFNMed	distance between the family and the nearest medical service (km)	17,702	1.422	2.35
IOPGDP	proportion of the output value of the industry in GDP (%)	112	48.83	9.35
CURIndus	comprehensive utilization rate of industrial solid waste (%)	112	81.76	22.72

Note: PHRP: abbreviations of perception of health risk; AHIncome: abbreviations of annual household income (CNY); DFNMed: abbreviations of distance between the family and the nearest medical service (km); IOPGDP:abbreviations of proportion of the output value of the industry in GDP (%); CURIndus: abbreviations of comprehensive utilization rate of industrial solid waste (%).

**Table 3:** Results of ordinal logistic regression analysis.

	PSM	Unmatched	PSM	Unmatched	PSM	Unmatched
	(1)	(2)	(3)	(4)	(5)	(6)
Time	−0.155 ** (0.045)	−0.202 *** (0.052)	−0.075 (0.052)	−0.135 ** (0.056)	−0.090 * (0.054)	−0.154 *** (0.059)
Airten	−0.156 *** (0.047)	−0.228 *** (0.052)	−0126 ** (0.049)	−0.152 ** (0.052)	−0.091 * (0.054)	−0.068 (0.059)
Time*Airten	−0.054 (0.050)	−0.009 (0.057)	−0.102 * (0.058)	−0.047 (0.062)	−0.122 ** (0.060)	−0.065 (0.064)
Age			0.039 *** (0.001)	0.038 *** (0.001)	0.039 *** (0.001)	0.039 *** (0.001)
DFNMed			0.038 *** (0.006)	0.040 *** (0.007)	0.038 *** (0.007)	0.040 *** (0.007)
LnAHIncome			−0.366 *** (0.016)	−0.367 *** (0.017)	−0.361 *** (0.017)	−0.360 *** (0.017)
CURIndus					−0.007 *** (0.001)	−0.007 *** (0.001)
IOPGDP					−0.010 *** (0.002)	−0.010 *** (0.002)
Individual FE	No	No	No	No	Yes	Yes
*N*	15,842	17,766	15,842	17,766	15,842	17,766
Pseudo R^2^	0.0017	0.0018	0.0507	0.0505	0.0545	0.0543

Note: Standard errors are reported in parentheses. *, **, and *** indicate significance at the 10%, 5%, and 1% levels, respectively. PSM: Propensity Score Matching.

**Table 4:** Results of different time window ordinal logistic regression analysis.

Time Window (Months)	1	2	3	4	5	6
Airten	−0.031 (0.088)	0.035(0.087)	−0.062 (0.101)	−0.049 (0.094)	−0.056 (0.079)	0.013 (0.116)
Time	−0.123 * (0.064)	−0.122 * (0.064)	0.119 * (0.064)	−0.098 (0.065)	−0.138 ** (0.063)	−0.162 *** (0.063)
Time*Airten	−0.219 ** (0.099)	−0.024 (0.101)	0.044 (0.112)	0.105 (0.118)	−0.055 (0.092)	−0.282 ** (0.132)
OR of DID coffe.	0.803	1.023	0.957	1.111	0.947	0.754
Control Var.	Yes	Yes	Yes	Yes	Yes	Yes
Individual FE	Yes	Yes	Yes	Yes	Yes	Yes
*N*	3960	3908	3578	3536	4360	3158
Pseudo R^2^	0.0536	0.0520	0.0667	0.0621	0.0525	0.0573

Note: Standard errors are reported in parentheses. *, **, and *** indicate significance at the 10%, 5%, and 1% levels, respectively. Nubers from 1–6 in the table mean the number of months of time window. OR means odds ratio. Control Var. means control variables. FE means fixed effect. *N* means the number of the variables. R^2^ means the regression square of the model.

**Table 5:** Ordinal logistic results of heterogeneity analysis in different regions of China.

	Full Sample			Subsample of 1-Month Time Window			Subsample of 6-Month Time Window		
	Western	Central	Eastern	Western	Central	Eastern	Western	Central	Eastern
	(1)	(2)	(3)	(4)	(5)	(6)	(7)	(8)	(9)
Time*Airten	0.193 (0.143)	−0.069 (0.111)	−0.221 ** (0.089)	0.023 (0.221)	−0.109 *** (0.234)	−0.056 (0.131)	0.182 (0.218)	0.024 (0.031)	−0.584 ** (0.249)
Control Var.	Yes	Yes	Yes	Yes	Yes	Yes	Yes	Yes	Yes
Individual FE	Yes	Yes	Yes	Yes	Yes	Yes	Yes	Yes	Yes
*N*	4398	3484	7114	850	1090	2238	879	1022	1596
Pseudo R^2^	0.0581	0.0484	0.0443	0.0801	0.0719	0.0410	0.0759	0.0520	0.0573

Note: Standard errors are reported in parentheses. **, and *** indicate significance at the 5%, and 1% levels, respectively.

**Table 6:** Ordinal logistic results of heterogeneity analysis of gender.

	Full Sample		Subsample of 1-Month Time Window		Subsample of 6-Month Time Window	
	Male	Female	Male	Female	Male	Female
	(1)	(2)	(3)	(4)	(5)	(6)
Time*Airten	−0.112 (0.089)	−0.122 (0.082)	−0.318 ** (0.139)	−0.230 * (0.132)	−0.143 (0.187)	−0.477 *** (0.184)
Control Var.	Yes	Yes	Yes	Yes	Yes	Yes
Individual FE	Yes	Yes	Yes	Yes	Yes	Yes
*N*	7498	8808	2094	2432	1714	1974
Pseudo R^2^	0.0560	0.0578	0.0523	0.0591	0.0593	0.0591

Note: Standard errors are reported in parentheses. *, **, and *** indicate significance at the 10%, 5%, and 1% levels, respectively.

**Table 7:** Ordinal logistic results of heterogeneity analysis of different ages.

	Full Sample		Subsample of 1-Month Time Window		Subsample of 6-Month Time Window	
	Age 18–55	Age > 55	Age 18–55	Age > 55	Age 18–55	Age > 55
	(1)	(2)	(3)	(4)	(5)	(6)
Time*Airten	−0.079 (0.072)	−0.247 ** (0.113)	−0.268 ** (0.118)	−0.266 (0.172)	−0.142 (0.150)	−0.821 *** (0.293)
Control Var.	Yes	Yes	Yes	Yes	Yes	Yes
Individual FE	Yes	Yes	Yes	Yes	Yes	Yes
*N*	11,290	4362	3152	1210	2608	942
Pseudo R^2^	0.0427	0.0259	0.0435	0.0269	0.0468	0.0265

Note: Standard errors are reported in parentheses. **, and *** indicate significance at the 5%, and 1% levels, respectively.

**Table 8:** Ordinal logistic regression results of different PSM matching methods.

	Neighbor Matching	Kernel Matching	Radius Matching
Time*Airten	−0.081 ** (0.038)	−0.122 ** (0.060)	−0.126 ** (0.058)
YesControl Var.	Yes	Yes	Yes
Individual FE	Yes	Yes	Yes
*N*	15,590	15,842	16,152
Pseudo R^2^	0.0580	0.0545	0.0556

Note: Standard errors are reported in parentheses ** indicates significance at the 5% level.

**Table 9:** Ordinal logistic regression results of further robustness test.

	Sample Indentation	Replace the Control Variables	Add Other Control Variables
	(1)	(2)	(3)
Time*Airten	−0.122 ** (0.0599)	−0.124 ** (0.060)	−0.126 ** (0.064)
Control Var.	Yes	Yes	Yes
Individual FE	Yes	Yes	Yes
*N*	16,304	16,294	14,772
Pseudo R^2^	0.0543	0.0543	0.0572

Note: Standard errors are reported in parentheses. ** indicates significance at the 5% level.

## Data Availability

Data can be downloaded at: https://github.com/fanyy07/allresearchdata.git (accessed on 16 December 2021).
